# Improvement in Depression Symptoms Measured by Montgomery-Åsberg Depression Rating Scale and Quick Inventory of Depressive Symptomatology-Self Rated Items after Randomised Double-blind COMP360 Psilocybin Therapy for Treatment-resistant Depression

**DOI:** 10.1192/j.eurpsy.2023.273

**Published:** 2023-07-19

**Authors:** G. M. Goodwin, L. Marwood, S. Mistry, A. Nowakowska, H. Simmons, J. Tsai, S. Williams, M. B. Young, E. Malievskaia

**Affiliations:** COMPASS Pathways, London, United Kingdom

## Abstract

**Introduction:**

COMP360 is a synthetic, proprietary, purified form of psilocybin in development for treatment-resistant depression (TRD) with FDA Breakthrough Therapy designation. In a recent phase IIb study, COMP360 psilocybin 25mg was superior to 1mg on change from baseline (CFB) to Week 3 on the Montgomery-Åsberg Depression Rating Scale (MADRS) total score (primary efficacy endpoint), when administered alongside psychological support. Quick Inventory of Depressive Symptomatology-Self Rated (QIDS-SR _16_) total score (exploratory efficacy endpoint) showed similar results.

**Objectives:**

To analyse changes in specific depression symptoms after psilocybin treatment in the aforementioned study, as measured by individual item scores on the MADRS and QIDS-SR _16_ (range 0-6 and 0-3).

**Methods:**

Participants with TRD were randomised to single doses of psilocybin 25mg (n=79), 10mg (n=75), or 1mg (n=79). A remote, blinded rater assessed the MADRS at Baseline, Day 2 (the day post-psilocybin), and Weeks 1, 3, 6, 9, and 12. The QIDS-SR _16_ was self-rated at Baseline, Day 1, Day 2, and Weeks 1, 2, 3, 6, 9, and 12. At each time point, descriptive statistics were calculated for each MADRS and QIDS-SR _16_ individual item score.

**Results:**

At Week 3, MADRS items with the largest differences in mean CFB in the 25mg arm were Inability to Feel, Apparent Sadness, Lassitude, and Reported Sadness. Greater improvement in the 25mg arm was apparent from Day 2 and remained to Week 12 (Lassitude remained to Week 6 only). On the QIDS-SR _16_, the item with the largest difference in mean CFB at Week 3 in the 25mg arm was in Feeling Sad and remained evident to Week 12 (Table 1).

**Table 1.** 

**Item (mean CFB at Week 3 [standard deviation])**	**Psilocybin 25mg**	**Psilocybin 10mg**	**Psilocybin 1mg**
**MADRS - Inability to Feel**	-1.8 [1.81]	-0.9 [1.54]	-0.8 [1.61]
**MADRS - Apparent Sadness**	-1.7 [1.94]	-1.1 [1.60]	-0.9 [1.62]
**MADRS - Lassitude**	-1.6 [1.81]	-1.2 [1.83]	-0.8 [1.58]
**MADRS - Reported Sadness**	-1.6 [1.95]	-1.0 [1.52]	-0.6 [1.53]
**QIDS-SR**_**16**_ **- Feeling Sad**	-1.1 [1.08]	-0.8 [1.07]	-0.4 [0.91]

**Conclusions:**

A single administration of COMP360 psilocybin therapy rapidly and dose-relatedly improved symptoms of depressed mood and anhedonia – the two key symptoms of depression. As anhedonia is predictive of poorer treatment response, and improvements in anhedonia correlate with improvements in functioning, it is important to understand the impact of treatments on this symptom.

**Disclosure of Interest:**

G. Goodwin Shareolder of: COMPASS Pathways, P1Vital, and P1Vital products , Employee of: COMPASS Pathways, L. Marwood Shareolder of: COMPASS Pathways, Employee of: COMPASS Pathways, S. Mistry Employee of: COMPASS Pathways, A. Nowakowska Employee of: COMPASS Pathways, H. Simmons Employee of: COMPASS Pathways, J. Tsai Employee of: COMPASS Pathways, S. Williams Employee of: COMPASS Pathways, M. Young Shareolder of: COMPASS Pathways, Employee of: COMPASS Pathways, E. Malievskaia Employee of: COMPASS Pathways

